# Clinical outcomes, complications, and survivorship for unicompartmental knee arthroplasty versus total knee arthroplasty in patients aged 80 years and older with isolated medial knee osteoarthritis: a matched cohort analysis

**DOI:** 10.1007/s00402-023-04916-9

**Published:** 2023-05-27

**Authors:** Riccardo D’Ambrosi, Chiara Ursino, Ilaria Mariani, Nicola Ursino, Matteo Formica, Antonia F. Chen

**Affiliations:** 1 IRCCS Istituto Ortopedico Galeazzi, Milan, Italy; 2 Department of Biomedical Sciences for Health, University of Milan, Milan, Italy; 3 Orthopaedic Clinic, IRCCS Hospital Policlinico San Martino, Genova, Italy; 4 DISC - Department of Surgical Sciences and Integrated Diagnostics, University of Genova, Genova, Italy; 5 Institute for Maternal, Child Health IRCCS Burlo Garofolo, Trieste, Italy; 6 Department of Orthopaedics, Brigham and Women’s Hospital, Harvard Medical School, Boston, MA USA

**Keywords:** Unicompartmental knee arthroplasty, Octogenarian, Total knee arthroplasty, Survivorship, Matched pair analysis

## Abstract

**Purpose:**

The primary goal of this study is to compare clinical outcomes, complication rate, and survivorship in octogenarians who underwent total knee arthroplasty (TKA) or unicompartmental knee arthroplasty (UKA) by performing a matched cohort analysis.

**Methods:**

We analyzed 75 medial UKAs performed by a single experienced surgeon. The included cases were matched with 75 TKAs performed during the same study period. Potential TKA matches used identical exclusion criteria. UKAs were age-, gender-, and body mass index (BMI)-matched at the rate of 1 UKA to 1 TKA from our departmental database. Clinical evaluation included the visual analog scale for pain, range of motion (ROM—flexion and extension), Knee Society Score (KSS), and Oxford Knee Score (OKS). Each patient was clinically evaluated on the day before the surgery (*T*_0_) and at two follow-ups at least 12 months (*T*_1_) and 24 months (*T*_2_) after the surgery. For the survivorship, revision was defined as failure of the implant (periprosthetic joint infection, periprosthetic fracture, or aseptic loosening), and survival was based on implant revision or patient death. Undesirable clinical developments that were not present at baseline or that increased in severity after treatment were classified as adverse events.

**Results:**

The mean age at the time of the surgery was 82.1 ± 1.9 years for UKA and 81.5 ± 1.8 years for TKA (*p* = 0.06). The two groups differed in regard to surgical time (UKA 44.9 ± 7.2 min; TKA 54.4 ± 11.3 min; *p* < 0.001); furthermore, the UKA group showed better function (ROM; flexion and extension) than the TKA group at each follow-up time point (*p* < 0.05). Both groups reported a significant improvement in all clinical scores (KSS and OKS) when compared with their preoperative status (*p* < 0.05), while no differences were found between the groups at each follow-up (*p* > 0.05). The UKA group reported 7 (9.3%) failures, while TKA reported 6 failures. There were no survival differences between the groups (*T*_1_: *p* = 0.2; *T*_2_: *p* = 0.5). Overall complication rate was 6% in the UKA group versus 9.75% in TKA (*p* = 0.2).

**Conclusion:**

The UKA and TKA patients had similar clinical outcomes, post-operative range of motion, and survivorship in octogenarians with medial knee osteoarthritis, with comparable complication rate. Both the surgical procedures may be considered in this patient population, but further long-term follow-up is needed.

**Level of evidence:**

Level III.

**Supplementary Information:**

The online version contains supplementary material available at 10.1007/s00402-023-04916-9.

## Introduction

Unicompartmental knee arthroplasty (UKA) can represent an alternative to total knee arthroplasty (TKA) for older patients (aged 80 years and older) with medial unicompartmental osteoarthritis (OA) of the knee [10]. Following TKA, older patients may have higher rates of medical and surgical complications than younger patients (< 65 years) due to more associated comorbidities and a lower tolerance for hemodynamic alterations [30]. UKA has been proven to have less problems than TKA when carried out on carefully chosen patients to restore knee kinematics and overall function more effectively [20, 31], and it is also less expensive [34, 36]. Historically, the implementation of UKA has been diminished by concerns about mechanical loosening and the necessity for revision [25]. With implant survivability currently averaging between 95 and 98% at 3 years [24], computer-navigated and robot-assisted surgeries, together with a better implant design, may have contributed to recent improvements in the UKA results. The age of 80 years has been identified as a significant threshold at which patients deserve additional evaluation and may have a higher risk of developing adverse outcomes for surgeries including spinal fusion, total shoulder arthroplasty, and revision total hip arthroplasty [4, 5, 12, 41]. More medical comorbidities, a reduced cardiac reserve, and greater difficulties in maintaining balance are common in older patients [44, 45]. To address the constraints brought on by their knee-joint arthritic pain, patients are prone to opt for partial and whole knee-joint arthroplasty, as it allows them to live longer and keep a high level of function and independence [9, 10]. Despite these advantages, UKA has been documented in literature to have higher revision rates than TKA; however, these results are often observed in younger patients (< 65 years) who may be more active, but the results may not apply to patients aged 80 years and older with potentially lower activity levels and life expectancy [15, 17, 42]. For example, according to the Kozinn and Scott criteria, the best candidates for cemented UKA were patients older than 60 years of age who are less physically active [23]. However, literature comparing UKA with TKA in a population of patients aged 80 years and older with isolated medial compartment OA is still scarce with conflicting results [15, 17, 42]. Thus, the primary goal of this study is to compare clinical outcomes and survivorship in UKA versus those in TKA in patients with isolated medial compartment OA and who are aged 80 years and older by performing a matched pair analysis.

## Materials and methods

The institutional review board approval was received to retrospectively review 90 medial UKAs consecutively performed by a single experienced surgeon. In total, 15 UKAs were excluded for the following reasons: 4 cases had previous osteotomies, 3 had rheumatoid arthritis, and 8 did not have a minimum 2-years follow-up.

The remaining 75 medial UKAs were matched with 75 TKAs performed during the same study period. Potential TKA matches used identical exclusion criteria. UKAs were age-, gender-, and body mass index-matched at a rate of 1 UKA to 1 TKA from our departmental database.

Inclusion criteria were a minimum 24-month follow-up, UKA or TKA performed by a single surgeon, and completion of follow-up evaluations.

Exclusion criteria were follow-up less than 24 months, revision surgery, rheumatoid arthritis, fixed varus deformity, previous osteotomy, or flexion deformity > 15°. Inclusion criteria for the TKA cohort were primary and traumatic isolated medial OA that met the criteria of UKA, but, for which, TKA was elected.

The primary indication was severe OA, with at least Kellgren–Lawrence grade 3 or post-traumatic arthritis only in the medial compartment [22]. In all patients, the anterior cruciate ligament and the medial and lateral collateral ligaments were functionally intact, the varus deformity was manually correctable, and there was no evidence of OA in the lateral compartment [43]. OA of the patellofemoral joint was not considered to be a contraindication, unless there was a deep eburnation or bone grooving on the medial facet of the patella (Outerbridge grade IV) [38].

All the procedures involving human participants in this study followed the ethical standards of the institutional and/or national research committee, as well as the 1964 Helsinki Declaration and its later amendments or comparable ethical standards. The study followed the STROBE checklist for cohort studies [8]. Finally, informed consent was obtained from all the participants.

### Surgical procedure and clinical protocol

#### UKA

All UKAs were performed with the same minimally invasive surgical approach and were mobile bearing using the Oxford Microplasty instrumentation (Zimmer-Biomet, Warsaw, Indiana, USA). All the patients were placed supine on a standard operating table after administering spinal anesthesia. A tourniquet was applied to the proximal thigh on the operative side and inflated to 300 mm Hg. The operative leg was placed in a thigh support, with the hip flexed to approximately 30° and the leg hanging. A midline incision was made, followed by a small medial parapatellar incision. The patella was not subluxed to avoid damage to the synovial reflections of the suprapatellar pouch. The margins of the medial tibial condyle were exposed and cleared ensuring that too much soft tissue is released. The medial meniscus was removed. Osteophytes were removed from the tibia, femur, and intercondylar notch. A routine inspection of the patellofemoral and lateral compartments was conducted to ensure that each patient had isolated medial knee OA. The anterior cruciate ligament (ACL) was also intact in all patients. First, the tibial cut was made sagittally as close to the ACL insertion as possible. However, precautions were taken not to cut the ACL fibers. The saw was placed parallel to the anatomical axis of the tibia and not tilted medially, laterally, anteriorly, or posteriorly. Then, the femoral cuts were made using the intramedullary guide [28].

#### TKA

The same surgeon performed all the TKAs with a standard medial parapatellar approach and without patellar eversion. Tibial resection was performed with an extramedullary guide, and distal femoral resection was performed with an intramedullary guide. All the patients received a cemented, posterior-stabilized implant (Vanguard; Zimmer-Biomet, Warsaw, IN). Intraoperatively, the patella was not resurfaced, and patelloplasty was routinely performed for all the patients, which included the removal of osteophytes, smoothing of the patellar articular surface, and denervation of the peripheral patellar using electrocautery [14].

The surgical time was defined as the time from the incision to closure. Both the patient groups followed the same rehabilitation protocol, which involved passive mobilization on the day of the surgery. On post-operative day 1, patients started active progressive mobilization of the joint and performed assisted walking with a walker or two crutches. Gradually, patients increased their weight and continued with isometric muscle toning exercises [19].

### Clinical evaluation

Demographic data, including age, sex, and BMI data on the side of operation and surgical time were collected. All the clinical assessments were performed by two independent clinicians who were not involved in the index surgery. The clinical evaluation entailed the visual analog scale (VAS) [40] for pain and range of motion (ROM—flexion and extension), which was assessed using a digital inclinometer that is the most accurate method of knee-angle measurement [16]. Patient-reported outcome measurements (PROMs), including the Knee Society Score (KSS) and Oxford Knee Score (OKS) [11, 32], were measured. Each patient was clinically evaluated on the day before the surgery (*T*_0_) and at two consecutive follow-ups at least 1 year (*T*_1_) and 2 years (*T*_2_) after the surgery.

### Survivorship

Revision was defined as failure of the implant (periprosthetic joint infection [PJI], periprosthetic fracture, or aseptic loosening), and survival was based on implant revision or patient death. Patient deaths were confirmed by contacting relatives. PJI was diagnosed according to the New Definition for Periprosthetic Joint Infection: From the Workgroup of the Musculoskeletal Infection Society [33]. Periprosthetic fracture was defined as tibia or femur fractures occurring within 15 cm from the joint line or 5 cm from the endomedullary stem, if present [2]. Patients were classified as having aseptic loosening, if they had symptoms including pain, instability, or swelling; had radiographic evidence of loosening; and did not meet the definition for PJI [6].

### Complications and adverse events

Undesirable clinical developments that were not present at baseline or that increased in severity after the treatment were classified as Adverse Events. Major complications included deep infection of implant, vascular injury, myocardial infarction, fast atrial fibrillation, stroke, pulmonary embolism, and cardiac arrest. Minor complications included superficial wound infection, acute retention of urine, deep vein thrombosis, pneumonia, and urinary tract infection [18].

### Statistical analysis

An estimated sample of 130 subjects, 65 for each group, was required to compare the VAS for pain between UKA and TKA with a two-sided Wilcoxon–Mann‒Whitney test, assuming a mean difference of 3 points, a standard deviation of 1.5 for both groups, 5% alpha, and 95% power. Given the same parameters, this sample also had 99% power to detect a pre–post difference using a Wilcoxon signed-rank test, assuming a correlation of 0.30 between measurements. Additional subjects were recruited to ensure statistical significance in case of adverse events.

Summary statistics were presented as the means and standard deviation (SD) or absolute frequencies and percentages. Having tested the distribution of continuous variables, a Student’s *t*-test or chi-square test for categorical variables was performed to assess preoperative differences between the UKA and TKA groups. To test score differences between the groups, a Student’s *t*-test was used to evaluate intergroup differences at each follow-up. Second, to assess differences in time in each group for each score, a linear mixed model was performed, since it takes into account correlations among repeated measures and tests the covariance structure. Autoregressive compound symmetry, with either homogeneous or heterogeneous variances, and unstructured covariance structures were tested. The best covariance structure was evaluated for each score using the likelihood-ratio test and Akaike information criterion. Bonferroni adjustment was applied for multiple comparisons. The Cox regression model was performed using failure as an independent variable and group as a covariate. All the tests were two sided, and *p* < 0.05 was considered statistically significant. Statistical analyses were conducted in R version 4.1.1 [35].

## Results

### Demographics

The mean age at surgery was 82.1 ± 1.9 years for UKA and 81.5 ± 1.8 years for TKA (*p* = 0.06); there were 59 female patients (78.7%) in the UKA group and 55 (72.9%) female patients in the TKA group (*p* = 0.45). The TKA group had a longer follow-up time than UKA (*p* < 0.001). Detailed results are reported in Table 1.

**Table 1:** Demographics of the patient population

	UKA *n* = 75 Mean ± SD	TKA *n* = 75 Mean ± SD	*p* value
Age (years)	82.1 ± 1.9	81.5 ± 1.8	0.06
Body Mass Index (kg/m^2^)	26.9 ± 3.5	28.0 ± 4.1	0.07
Sex*, n (%)*			
Female	59 (78.7)	55 (73.3)	0.45
Male	16 (21.3)	20 (26.7)	
Side*, n (%)*			
Right	34 (45.3)	44 (58.7)	0.1
Left	42 (54.7)	31 (41.3)	
Surgical time (min)	44.9 ± 7.2	54.4 ± 11.3	< 0.001*
Follow-up (months)			
1-year	12.9 ± 0.8	12.9 ± 1.4	0.9
2-year	36.4 ± 10.5	44.6 ± 10.8	< 0.001*

*TKA* total knee arthroplasty, *UKA* unicompartmental knee arthroplasty, *VAS* visual analog scale

^*^Statistically significant difference (*p* < 0.05)

### Group comparison

The two groups differed in regard to surgical time (UKA: 44.9 ± 7.2 min; TKA: 54.4 ± 11.3 min; *p* < 0.001); furthermore, the UKA group showed better function (ROM; flexion and extension) than TKA at each follow-up time point (*p* < 0.05). Both the groups reported a significant improvement in all clinical scores (KSS and OKS) when compared with their preoperative status (p < 0.05), while no differences were found between the two groups at each follow-up (*p* > 0.05) (Table 2).

**Table 2:** Clinical comparison between groups at each post-operative follow-up

	UKA	TKA	Group comparison	Time comparison adjusted *p* value			
	Mean ± SD	Mean ± SD	*p* value	UKA		TKA	
VAS pain							
Preop	7.4 ± 1.3 (*n* = 75)	7.4 ± 1.2 (*n* = 75)	0.9	Preop	1-year	Preop	1-year
1 year	1.7 ± 1.2 (*n* = 74)	2.0 ± 1.3 (*n* = 71)	0.2	< 0.001*	–	< 0.001*	–
2 years	1.4 ± 0.9 (*n* = 68)	1.5 ± 0.9 (*n* = 69)	0.5	< 0.001*	0.1	< 0.001*	0.02*
Flexion (°)							
Preop	96.7 ± 8.2 (*n* = 75)	90.1 ± 9.0 (*n* = 75)	< 0.001*	Preop	1-year	Preop	1-year
1 year	116.7 ± 5.3 (*n* = 74)	113.9 ± 7.3 (*n* = 71)	0.01*	< 0.001*	-	< 0.001*	-
2 years	117.6 ± 4.0 (*n* = 68)	119.1 ± 4.1 (*n* = 69)	0.03*	< 0.001*	0.3	< 0.001*	< 0.001*
Extension							
Preop	3.8 ± 3.1 (*n* = 75)	5.1 ± 2.9 (*n* = 75)	0.01*	Preop	1-year	Preop	1-year
1 year	0.5 ± 1.6 (*n* = 74)	1.8 ± 2.8 (*n* = 71)	0.001*	< 0.001*	–	< 0.001*	–
2 years	0.4 ± 1.4 (*n* = 68)	1.1 ± 2.2 (*n* = 69)	0.04*	< 0.001*	0.7	< 0.001*	0.08
Knee Society Score							
Preop	36.8 ± 8.8 (*n* = 75)	36.2 ± 8.7 (*n* = 75)	0.6	Preop	1-year	Preop	1-year
1 year	90.2 ± 6.6 (*n* = 74)	89.7 ± 6.9 (*n* = 71)	0.7	< 0.001*	–	< 0.001*	–
2 years	91.0 ± 4.9 (*n* = 68)	91.2 ± 5.6 (*n* = 69)	0.9	< 0.001*	0.4	< 0.001*	0.2
Oxford Knee Score							
Preop	21.5 ± 3.7 (*n* = 75)	20.8 ± 3.8 (*n* = 75)	0.2	Preop	1-year	Preop	1-year
1 year	43.6 ± 2.1 (*n* = 74)	43.8 ± 2.0 (*n* = 71)	0.4	< 0.001*	–	< 0.001*	–
2 years	43.9 ± 1.7 (*n* = 68)	44.0 ± 2.3 (*n* = 69)	0.8	< 0.001*	0.3	< 0.001*	0.7

*TKA* total knee arthroplasty, *UKA* unicompartmental knee arthroplasty, *VAS* visual analog scale

^*^Statistically significant difference (*p* < 0.05)

### Failures and death

The UKA group reported 7 (9.3%) failures, of which one was during the first year and 6 before the 24-month follow-up point. The TKA group reported six failures, of which four were during the first year and two before the 24-months follow-up. All the deceased patients died for reasons not related to the arthroplasty, as confirmed over phone calls by relatives.

There were no survival differences between the groups (*T*_1_: *p* = 0.2; *T*_2_: *p* = 0.5). Failure details are reported in Table 3.

**Table 3:** Failures and deaths for each group and related time point

UKA group		TKA group	
Time point	Failure	Time point	Failure
6 months	Death	2 months	Revision for deep infection
16 months	Revision for aseptic loosening	3 months	Periprosthetic fracture
18 months	Revision for aseptic loosening	8 months	Death
22 months	Bearing dislocation	10 months	Death
25 months	Revision for aseptic loosening	15 months	Death
26 months	Death	20 months	Death
30 months	Death		

*UKA* Unicompartmental Knee Arthroplasty, *TKA* Total Knee Arthroplasty

## Complications

In total, two patients (41.5%) in the UKA group suffered from major complications and three from the control TKA group (2.25%; *p* = 0.7). Overall complication rate was 6% in the UKA group versus 9.75% in TKA (*p* = 0.2). Minor complication rates were 4.5% versus 7.5% (UKA versus TKA; *p* = 0.3). All complications, major and minor, are charted in Table 4.

**Table 4:** Post-op complications

Post-op complications		UKA (75)	TKA (75)	*p* value
		Number (%)	Number (%)	
Major complications	Deep implant infection	0 (0%)	1 (0.75%)	
	Vascular injury	1 (0.75%)	1 (1.5%)	
	Myocardial infarction	0 (0%)	0 (0%)	
	Fast atrial fibrillation	0 (0%)	0 (0%)	
	Stroke	0 (0%)	0 (0%)	
	Pulmonary embolism	1 (0.75%)	1 (0.75%)	
	Cardiac Arrest	0 (0%)	0 (0%)	
Minor complications	Superficial wound infection	1 (0.75%)	2 (1.5%)	
	Acute retention of urine	0 (0%)	1 (0.75%)	
	Deep vein thrombosis	4 (3%)	6 (4.5%)	
	Pneumonia	1 (0.75%)	0 (0%)	
	Urinary tract infection	0 (0%)	1 (0.75%)	
Major complication rate		2 (1.5%)	3 (2.25%)	0.7
Minor complication rate		6 (4.5%)	10 (7.5%)	0.3
Overall complication rate		8 (6%)	13 (9.75%)	0.2

*UKA* Unicompartmental Knee Arthroplasty, *TKA* Total Knee Arthroplasty

## Discussion

There have been limited studies comparing UKA and TKA in octogenarians [31, 37]. Our study found that both UKA and TKA in this patient population improved patient function as measured by PROMs, with decreased pain in both the patient cohorts and similar rates of failure, deaths, and complications.

In our study, patients who underwent UKAs showed a slightly but significantly shorter surgical time than those who underwent TKAs (UKA 45.0 ± 7.4 min; TKA 53.3 ± 10.8 min), and these findings can play a crucial role in treating older patients with systematic diseases.

Morcos et al. [27] evaluated the influence of operating time on complications and readmission within 30 days of TKA, concluding that an operating time of 90 min or more may be associated with an increase in the chances of 30-day complications and readmissions following TKA. Similarly, Cregar et al. [7] revealed a positive correlation between increased operative times and short-term post-operative complication rates after UKA. Thus, for the older patient population with increased comorbidities in this study, shorter operative time may be beneficial for outcomes and decreasing complications.

In this study, both the types of implants showed significant improvements in all the clinical scores, which is consistent with the literature where PROMs were not significantly different between UKA and TKA.

Often, elderly patients have a more sedentary lifestyle and less functional demand for knee arthroplasty than younger patients, which may reduce the risk of aseptic loosening. Although, according to the literature, TKA survivorship is greater than UKA, our data suggest that UKA may be considered an appropriate option for this patient population, since there was no difference in survivorship between the groups. This corresponds with some studies comparing UKA and TKA survivorship in patients older than 75 years. The study by Siman et al. [37] found almost no difference in 5-years survivorship estimates for UKA (98.3%) and TKA (98.8%).

Ode et al. [31] performed a retrospective control study comparing complication rates in elderly patients receiving UKA with those receiving TKA. At a mean, follow-up was 32 months for UKA and 34 months for TKA. The complication rate was significantly lower with UKA (6.7% versus 25.6%), with no early mortality. Similarly, satisfaction rates were identical: 96% and 97%. Implant survivorship was also identical.

In recent years, several articles have analyzed the outcomes of prostheses (both UKA and TKA) in patients over 80 years of age with satisfactory results, as in our study. Trigueros-Larrea et al. [42] conducted a retrospective observational study in octogenarians comparing preoperative and post-operative KSS, Knee Society Function Score (KSFS), extension and flexion, and radiologic alignment. The mean patient survival was 67.4 months. Patients ≥ 80 years achieved clinical improvement after TKA. Comorbidities, not age, were found to be the burden for surgery in older patients. Goh et al. [15] analyzed the results of UKA in the extreme elderly (≥ 80 years) by comparing the functional and perioperative outcomes between octogenarians and age-appropriate controls undergoing UKA. With the exception of poorer Short Form-12 physical scores in octogenarians at 2 years (*p* = 0.03), there was no difference in final post-operative scores between the groups. The rates of complications, reoperations, readmissions, and emergency room visits were also similar. The 5-years survivorship was 97% in the control group and 93% in the octogenarian group (*p* = 0.15).

Moore et al. [26] used a large national surgical database to examine 30-days post-operative adverse events after UKA in octogenarians compared with those in non-octogenarians. The authors found a statistically significant increase in several adverse events within 30 days of the surgery for patients aged > 80 years when compared with patients < 80 years, namely, UKA in octogenarians was associated with significantly increased odds of short-term mortality, urinary tract infection, transfusion, prolonged hospital stay, and readmission.

Recently, D’Ambrosi et al. [10] compared clinical difference and survivorship between fixed and mobile bearing in octogenarians, finding no difference in patient-reported outcome measures, ROM, implant positioning, and survivorship.

The same authors analyzed survivorship and functional results in individuals aged 80 years and over who underwent TKA with cruciate-retaining (CR) or posterior-stabilized (PS) implants. The clinical trial demonstrated that CR and PS TKA had similar clinical outcomes in octogenarians with regard to knee function, post-operative knee pain, and other complications [9].

However, our patients demonstrated some slight difference in ROM between the two groups. This finding was also observed by other studies. In the 15-year results of a prospective randomized controlled trial by Newman et al. [29], UKA achieved higher degrees of flexion than TKA, and this was maintained at the 15-years follow-up. In contrast, in our cohort, we found higher flexion at the 2-years follow-up in the TKA group (UKA 117.59 ± 3.97; TKA 119.33 ± 4.07), which may not be clinically significant.

Both the groups reported a similar number of failures throughout the follow-up period. There were no revisions in the UKA group until one year after surgery, which is comparable to the findings in the literature. A recent study by Carlson et al. [3] demonstrated that the peak of failure after Oxford UKA occurred within three years, with a second peak at seven years. Ekthiari et al. [13] reported that survivorship at 1, 5, 10, and 15 years was 97.2%, 90.5%, 83.5%, and 81.9%, respectively, for patients who underwent UKA surgery for medial knee OA. The most common mode of failure for UKA is OA progression, but it should be taken into consideration that octogenarian patients may not have a long enough life expectancy for this longer-term failure to occur [39]. In our patient population, no patients failed due to secondary OA progression within two years. Most of TKA group failures were observed within one year of follow-up; historically, studies comparing failures and revisions in TKA and UKA patients have demonstrated a trend for greater revisions in UKA patients than in TKA patients. In the study by Arirachakaran et al. [1], the rate of revision in the UKA group was 3.2 times higher than that in the TKA group. In long-term studies, TKA has established a survivorship of 92–100%. In a large retrospective database analysis comparing UKA to TKA, Kaplan‒Meier survivorship at 5 and 10 years was 98% and 95%, respectively, for TKA and 95% and 90%, respectively, for UKA. Horikawa et al. [21] reported a cumulative revision rate greater for UKA (7%) than that for TKA (4%), and Kaplan‒Meier survivorship at 10 years was 84% for UKA and 92% for TKA. It is important to note that some of these studies included all the patients undergoing UKA, and these findings may not apply to older patients.

Our study had several limitations. First, this study was conducted at a single institution and may not be applicable to other patient populations. Second, it was a retrospective study of prospectively collected data with a potential inherent selection bias. Moreover, multiplane laxity measurements were not considered in our study as part of the clinical evaluation. Finally, this study only evaluated short-term follow-up of two years, and further long-term studies are needed to assess differences between these patient cohorts.

### Conclusion

UKA and TKA patients had similar clinical outcomes, post-operative range of motion, and survivorship in octogenarians with medial knee OA with a comparable complication rate. Both surgical procedures may be considered in this patient population, but further long-term follow-up is needed.

## Supplementary Information

Below is the link to the electronic supplementary material.

Supplementary file1 (XLSX 64 KB)

## Data Availability

Raw data have been submitted as supplementary material to the Journal.
